# Ontogenetic Pattern Changes of Nucleobindin-2/Nesfatin-1 in the Brain and Intestinal Bulb of the Short Lived African Turquoise Killifish

**DOI:** 10.3390/jcm9010103

**Published:** 2019-12-31

**Authors:** Alessia Montesano, Elena De Felice, Adele Leggieri, Antonio Palladino, Carla Lucini, Paola Scocco, Paolo de Girolamo, Mario Baumgart, Livia D’Angelo

**Affiliations:** 1 Department of Veterinary Medicine and Animal Production, University of Naples Federico II, 80137 Naples, Italy; alessia.montesano@leibniz-hki.de (A.M.); adele.leggieri@unina.it (A.L.); lucini@unina.it (C.L.); degirola@unina.it (P.d.G.); 2 Leibniz Institute on Aging–Fritz Lipmann Institute, 07745 Jena, Germany; mario.baumgart@leibniz-fli.de; 3 Leibniz Institute for Natural Product Research and Infection Biology–Hans Knöll Institute, 07745 Jena, Germany; 4 School of Bioscience and Veterinary Medicine, University of Camerino, 62032 Camerino, Italy; elena.defelice@unicam.it (E.D.F.); paola.scocco@unicam.it (P.S.); 5 Center for Advanced Biomaterials for Health Care, IIT@CRIB, Istituto Italiano di Tecnologia, 80125 Naples, Italy; a.palladino1986@gmail.com; 6 Stazione Zoologica Anton Dohrn, 80122 Naples, Italy

**Keywords:** Nesf-1, vertebrate, *Nothobranchius furzeri*, aging, brain-gut axis

## Abstract

Nesfatin-1 (Nesf-1) was identified as an anorexigenic and well conserved molecule in rodents and fish. While tissue distribution of NUCB2 (Nucleobindin 2)/Nesf-1 is discretely known in vertebrates, reports on ontogenetic expression are scarce. Here, we examine the age-related central and peripheral expression of NUCB2/Nesf-1 in the teleost African turquoise killifish *Nothobranchius furzeri*, a consolidated model organism for aging research. We focused our analysis on brain areas responsible for the regulation of food intake and the rostral intestinal bulb, which is analogous of the mammalian stomach. We hypothesize that in our model, the stomach equivalent structure is the main source of NUCB2 mRNA, displaying higher expression levels than those observed in the brain, mainly during aging. Remarkably, its expression significantly increased in the rostral intestinal bulb compared to the brain, which is likely due to the typical anorexia of aging. When analyzing the pattern of expression, we confirmed the distribution in diencephalic areas involved in food intake regulation at all age stages. Interestingly, in the rostral bulb, NUCB2 mRNA was localized in the lining epithelium of young and old animals, while Nesf-1 immunoreactive cells were distributed in the submucosae. Taken together, our results represent a useful basis for gaining deeper knowledge regarding the mechanisms that regulate food intake during vertebrate aging.

## 1. Introduction

Nucleobindin (NUCB) belongs to the family of calcium and DNA binding proteins and comprises two members, NUCB1 and NUCB2. In mammals, NUCB1 is expressed within the pituitary, liver, and kidney, where it regulates calcium homeostasis and G protein signaling [1,2], while NUCB2 is expressed in the appetite-controlling hypothalamic nuclei, such as the lateral area of the hypothalamus (LHA), paraventricular nucleus (PV), arcuate nucleus (ARC), supraoptic nucleus (SON), tractus solitarius nucleus (NTS), and dorsal nucleus of the vagus [3]. NUCB2 is post-translationally cleaved by prohormone convertases into one N-terminal fragment, Nesfatin-1 (residues 1–82), and two C-terminal peptides, Nesfatin-2 (residues 85–163) and Nesfatin-3 (residues 166–396) [4]. Only the mid-segment of Nesfatin-1 (Nesf-1) is considered the bioactive core, exercising anorexigenic effects [3,4,5]. In fact, Kohno and colleagues [6] showed that in PV and SON nuclei, which are immunoreactive (ir) to oxytocin and vasopressin, Nesf-1 positive neurons play a key role in the post-prandial regulation of food intake and peripheral metabolism. Hypothalamic neurons which co-expressed Nesf-1, antidiuretic hormone (ADH), corticotropin (CRH), and thyrotropin-releasing hormone (TRH) constitute an important network which regulates food intake. This network acts through the anorexigenic system of the melanocortin above all. It has been shown that the intracerebro-ventricular administration of both NUCB2 and Nesf-1 reduces food intake and body weight as well as increases sympathetic nerve activity and blood pressure in rats [3]. However, the use of anti-Nesf-1 antibodies is not enough to inhibit the sense of satiety induced by leptin: therefore, Nesf-1 is an anorexigenic molecule with a leptin-independent action in mammals [7]. As with many other central appetite regulators, NUCB2/Nesf-1 was also detected in peripheral tissues in different vertebrate models that had an important role in energy homeostasis, such as adipose tissue [8], pancreas [9,10], and in middle and lower segments of gastric mucosal glands [11], as well as in the submucosal layer of the duodenum [9]. Stengel and colleagues described NUCB2/Nesf-1 in the gastric X/A-like endocrine cells of the stomach and showed co-localization with the orexigenic hormone ghrelin in mammals. Moreover, expression levels of NUCB2/Nesf-1 in purified small endocrine cells of gastric mucosa have been reported to be 10-fold higher compared to brain levels [11]. Based on these indications, the stomach is considered one of the main sources of circulating NUCB2/Nesf-1 [12], supporting the hypothesis that NUCB2 is cleaved and Nesf-1 is produced at the gastric level [13].

In the last few years, several studies have described the distribution pattern of NUCB2/Nesf-1, also reporting the appetite regulatory effect in non-mammalian vertebrates. Particularly, in teleost fish, NUCB2 mRNA is widely expressed in central and peripheral tissues, mostly in the brain and gut. Similarly to mammals, the highest NUCB2/Nesf-1 mRNA expression was found in the gastrointestinal tract [14,15,16]. Nesf-1 ir-cells were found in the feeding regulatory nuclei of the hypothalamus and anterior intestine of the goldfish [14] and in the mucosal cellular layer of the anterior gastrointestinal tract in zebrafish [16]. NUCB2/Nesf-1 and ghrelin co-localize in the enteroendocrine cells of the anterior intestine [17] and in the hepatopancreas of goldfish [18], in the gut mucosal cells of zebrafish [16], and in intestinal enteroendocrine cells of pejerry [19]. Furthermore, the anorexigenic role of Nesf-1 seems to also be conserved in goldfish and Ya-fish, where central or peripheral administration of Nesf-1 induces feeding behavior suppression [14,15,17] as well as in Siberian sturgeon, where it acts through the cholecystokinin signal pathway [20,21].

Despite the fact that tissue distribution patterns are well described in vertebrates, few studies are dedicated to the determination of the expression of NUCB2 mRNA and Nesf-1 immunoreactivity during ontogenesis and postnatal development. Mohan and Unniappan [22] demonstrated that NUCB2/Nesf-1 immunoreactivity in the pancreas and gastrointestinal tract of rats increased from embryonic day 21 through to postnatal day 27, which is likely related to weaning. Senin and colleagues [23] confirmed and expanded previous data on gastroenteropancreatic tissues of rats by analyzing NUCB2/Nesf-1 expression from 2 until 8 weeks of age, corresponding to adulthood. However, there are no reports regarding age-related changes of NUCB2/Nesf-1 in the brain and stomach of any vertebrate. Here, we investigate the expression of Nesf-1 in the African turquoise killifish *Nothobranchius furzeri,* a well consolidated model organism for aging research. *N. furzeri* is the shortest-lived vertebrate that can be kept in captivity, with a lifespan of certain strains of 4 to 6 months in optimal laboratory conditions (6–10 times shorter than the lifespan of mice and zebrafish, respectively) [24]. In fact, *N. furzeri* is characterized by rapid growth, early sexual maturation [25,26], and the development of several biomarkers of aging during the short lifespan [27].

Thus, the aim of the present study is to investigate for the first time the age-related central and peripheral expression of NUCB2/Nesf-1 in *N. furzeri,* achieving deeper knowledge in food intake regulation during aging. Additionally, this study contributes to widely characterize the food intake regulation of the African turquoise killifish [28] and enrich data on neuropeptides that regulate food intake in fish [29,30,31,32].

## 2. Materials and Methods

### 2.1. Protocols and Ethics Statement

All experiments were performed on group-housed *N. furzeri* belonging to the long-lived strain MZM 04/10 (Leibniz Institute on Aging Friz-Lipmann Institute, Germany, Jena) at the following time points: 5 weeks post hatching (wph) (young-adult) and 27 wph (onset of aging-related features). Animal maintenance was performed as described [24]. Animals were bred and kept in FLI’s fish facility according to paragraph 11 of the German Animal Welfare Act. The protocols of animal maintenance were approved by the local authority in the State of Thuringia (Veterinaer- und Lebensmittelueberwachungsamt) with license number J-003798. Euthanasia and organ harvesting was performed according to paragraph 4 (3) of the German Animal Welfare Act and The Council of The European Union Directive of 22nd of September 2010 (2010/63/UE).

### 2.2. Animals and Tissue Preparation

Fish at the selected time point were euthanized at 10 a.m. with an overdose of anesthetics. Fish, without prior sedation, were placed in a buffered Tricaine methanesulfonate solution (MS-222, TricanePharmaq, Pharmaq) at a concentration of 1 mg/mL for approximately 5–10 min until no vital signs were observed (body and operculum movement, righting reflex), followed by decapitation. The whole heads, brains, and intestines were dissected and processed according to the experimental protocols. For RNA extraction, brains were immediately processed as described in Baumgart et al. 2014 [33]. For morphological analysis, the whole heads were opened by a small incision to allow penetration of a fixative and were fixed in paraformaldehyde (PFA, 4% in diethylpyrocarbonate treated phosphate saline buffer (PBS)) overnight (ON) at 4 °C and the brains were prepared the next day to maintain structural integrity. For cryostatic embedding, tissues were successively incubated in 20% and 30% sucrose solution ON at 4 °C, embedded in cryomount (Tissue-Tek^®^ O.C.T.™, Sakura Finetek USA Inc., Torrance, CA, USA), and frozen at −80 °C. Serial coronal sections of 14 µm thickness for the brain and sagittal sections of 16 µm for the intestine were cut with a Leica cryostat (Deerfield, IL, USA). For paraffin embedding, tissues were dehydrated in a graded ethanol series, embedded in paraffin, and serial coronal 7 µm thick sections were cut at the microtome.

### 2.3. Sequence Analysis

*N. furzeri* NUCB2 gene structure was recovered from the *Nothobranchius furzeri* Genome Browser–NFINgb [34], while human, mouse, and zebrafish sequences were recovered from the Ensembl Genome Browser [35]. The gene structure analysis was based on sequences retrieved by the Ensembl Genome Browser (Table S1). The evolutionary history was inferred using the Minimum Evolution method [36]. The optimal tree with the sum of branch length = 3.06014219 is shown. The tree is drawn to scale, with branch lengths in the same units as those of the evolutionary distances used to infer the phylogenetic tree. The evolutionary distances were computed using the Poisson correction method [37] and are in the units of the number of amino acid substitutions per site. The ME tree was searched using the Close-Neighbor-Interchange (CNI) algorithm [38] at a search level of 1. The Neighbor-joining algorithm [39] was used to generate the initial tree. This analysis involved 5 amino acid sequences. All ambiguous positions were removed for each sequence pair (pairwise deletion option). There were a total of 496 positions in the final dataset. Evolutionary analyses were conducted in MEGA X [40]. NUCB2 aminoacidic sequences were recovered from the National Center for Biotechnology Information–NCBI [41] and the alignment was performed using Clustal Omega [42]. Identity percentage among sequences was calculated with the Basic local alignment search tool—Blast [43].

### 2.4. RNA Extraction and Reverse Transcription of cDNA Synthesis

Homogenization of tissues was performed using a Tissue Lyzer II (Qiagen, Hilden, Germany) at 20 Hz for 2 to 3 rounds × 1 min [44]. Total RNA was quantized with a NanoDrop 1000 (PeqLab, Erlangen, Germany). Then, 500 ng of each sample was retro-transcribed in a total reaction volume of 20 µL using the QuantiTect^®^ Reverse Transcription Kit (Qiagen), following the supplier’s protocol. The cDNAs were diluted to a final volume of 200 µL with nuclease-free water (Qiagen) and stored at −20° C.

### 2.5. Quantitative Real Time PCR

Primers were designed with Primer3 tool [45]: forward and reverse primers were always located in two different exons. The primers that were used were summarized in Table S2. The correct amplicon size was verified by 1% agarose-gel electrophoresis. Real-time PCR reactions were performed in 20 μL volume with 1 μL diluted cDNA using the Quantitect^®^ SYBR Green PCR kit (Qiagen) following the manufacturer’s instructions. A cDNA pool was serially diluted (from 80 to 2.5 ng per reaction) and used to create standard as well as melting curves and to calculate amplification efficiencies for the primer pair prior to use for quantification. All reactions were performed in triplicates and negative (water) as well as genomic (without reverse transcriptase) controls were always included.

### 2.6. Statistical Analysis

We analyzed the expression levels NUCB2 mRNA in the whole brain and stomach of 22 animals in total at 5 wph (*n* = 12) and 27 wph (*n* = 10). Expression levels were analyzed by the ΔΔCt method and normalized to the housekeeping gene TATA box binding protein (TBP) (Table S2). Fold changes represent the normalized fold difference in expression levels relative to 5 weeks-old brain. T-test and *p*-value were calculated with Graphpad Prism among young and old brains and young and old rostral intestinal bulbs. Furthermore, T-test and p-value were also calculated for young brains versus young intestinal bulbs and old brains versus old intestinal bulbs.

### 2.7. Riboprobes Synthesis

mRNA probes to identify NUCB2B were synthesized by *in vitro* transcription (IVT). Oligonucleotide primers were designed using Primer3 software [45]. Reverse primer contained a T7-promotor sequence to allow direct IVT and the experiment was carried out by means of a DIG RNA labeling mix containing digoxigenin-11-dUTP (Roche, cat. 11277073910). Primers sequences that were used for this study are summarized in Table S2. Primers were diluted to a final concentration of 10 pM. A standard PCR was run to amplify the target region prior to IVT and the amplicon was checked by agarose electrophoresis. Alignment of the obtained sequences was performed by MEGA X software (Molecular Evolutionary Genetics Analysis from www.megasoftware.net) to validate the expected sequence of the amplicon. The concentration of the mRNA probe was measured using the Nanodrop^®^ (Thermo Scientific, Waltham, MA, USA) system.

### 2.8. In Situ Hybridization

In situ hybridization (ISH) experiments have been conducted on brain and intestine sections and by means of sterile solutions and materials. Dyethilpyrocarbonate (DEPC) was added to millipore water (1 mL/L) to inactivate RNases, shaken and autoclaved. All solutions that were used in the steps until probe revelation were made with RNase free water and RNAse free conditions were kept during all prehybridization and hybridization steps.

Sections were dried for 2 h at room temperature (RT), washed well in 1× DEPC/PBS, and treated with 10 µg/µL Proteinase K (Sigma–Aldrich) 1:200 in 1× DEPC/PBS for 10 min. Proteinase K was inactivated by two washes in 2 mg/mL glycine, 5 min each. Sections were post fixed in 4% PFA for 20 min and well washed in 1× DEPC/PBS at RT. Prehybridization was carried out in a hybridization solution containing 50% formamide, 0.5% SSC (Saline-Sodium Citrate), 500 µg. Heparin, 50 µg/mL yeast RNA, and 0.1% Tween 20 at 55 °C for 1 h. Probes were denatured for 10 min at −80 °C and sections were then incubated ON at 55 °C in hybridization solution containing antisense NUCB2B probe at a concentration of 2 ng/µL. Post hybridization washes were carried out at 55 °C as follows: 2 × 20 min in 1× SSC, 2 × 10 min in 0.5× SSC and then in PBS added with tween20 (PBT) at RT. The sections were blocked with a blocking solution (BS) containing 10% normal sheep serum heat inactivated and 0.5% blocking reagent (Roche, Basel, Switzerland, cat. 11096176001) for 1 h at RT. Sections were incubated ON at 4 °C in fresh blocking buffer containing a 1:2000 dilution of anti-digoxigenin Fab fragments conjugated with alkaline phosphatase (Roche, cat. 11093274910). After washing in PBS and levamisole (Vector Labs., Burlingame, CA, USA, SP-5000), the reaction was developed using Fast Red substrate (Roche, cat. 11496549001) under periodic inspection with an epifluorescence microscope. To stop the reaction, the sections were washed repeatedly in PBT and mounted with Fluoroshield Mounting Medium with DAPI (IBSC, Hermon, ME, USA, cat. AR-6501-0) as counterstaining for the nuclei.

### 2.9. Western Blot

Brain samples of young and old fish were extracted in RIPA buffer (Radio Immuno Precipitation Assay) with Lysis buffer (50 mM Tris–HCl pH 7.4, 1% Triton X-100, 0.25% Na-deoxycholate, 150 mM NaCl, 1 mM EDTA), containing protease inhibitors 2 mM phenylmethylsulfonyl fluoride (PMSF) and protease inhibitor cocktail (P8340; Sigma-Aldrich, St. Louis, MO, USA). Samples were homogenized with an Ultra-Turrax T25 (IKA Labortechnik, Staufer, Germany) at 13,500 rpm. Homogenates were centrifuged 15,000 rpm for 30 min at 4 °C; supernatants were collected separately and the protein concentration was determined with Bio-Rad dye protein assay (Bio-Rad Laboratories Inc., Hemel Hempstead, UK). For each sample, 20 µL of protein were boiled at 100 °C for 10 min in 20 µL of 2× loading buffer (50 mM Tris–HCl pH 6.8, 100 mM b-mercaptoethanol, 4% SDS, 0.1% blue bromophenol, 10% glycerol). Proteins were separated on a 12% SDS–polyacrylamide gel electrophoresis with 4% stacking gel in 1% Tris–glycine buffer (0.025 M Tris, 0.190 M glycine, and 0.1%SDS [pH 8.3]) in a miniprotean cell (Bio-Rad) at 100 V for 2 h. The separated proteins were electro-transferred on a nitrocellulose membrane with transfer buffer (48 mM Tris base, 39 mM glycine, 0.04% SDS, and 20% methanol [pH 8.5]) in a minitransfer cell (Bio-Rad) at 100 V at 4 °C for 1 h. Membranes were incubated at 4 °C for 1 h in blocking buffer containing 1% PBS and 0.05% Tween 20 with 5% dry non-fat milk and probed with polyclonal antibody raised in rabbit against Nesf-1 (H-003-24; Phoenix Pharmaceuticals; Belmont, CA, USA) and β-actin (A5060, Sigma, St. Louis, MO, USA) used as an internal marker. Primary antibody was diluted 1:2000 and incubated overnight at 4 °C, followed by incubation with the secondary goat anti-rabbit IgG (Sigma 1:3000) antibody for 1 h at RT. Signals were detected by chemoluminescence with the Pico Enhanced Chemiluminescence Kit (Pierce Chemical) with Chemidoc (Bio-Rad, Hercules, CA, USA). A pre-stained molecular-weight ladder (Novex Sharp Pre-Stained Protein Standard, Life Technologies, Monza, Italy) was used to determine protein size. Specificity was determined by pre-absorption of primary antibodies with their relative control peptides before western blotting.

### 2.10. Immunohistochemistry

Immunohistochemistry (IHC) was conducted on both paraffin and cryosections of brain and intestine. Paraffin slides were deparaffinized in xylene and rehydrated in progressively diluted alcohols, while cryosections were dried for 2 h at RT, placed in a bath of acetone 100% for 10 min at 4 °C, air-dried for a few minutes, and washed once in water and twice in 1× PBS. Then, both slides were treated for 30 min with 3% H_2_O_2_ and, after washing with 1× PBS, were incubated in normal goat serum (1:5 in 1× PBS) at RT for 30 min. Incubation with primary polyclonal antibody raised in rabbit against Nesf-1 (1:1000, H-003-24; Phoenix Pharmaceuticals; Belmont, CA, USA) was performed at 4 °C ON. Sections were rinsed in 1× PBS for 15 min and incubated with EnVision reagent (DAKO, K406511) for 30 min at RT. Immunoreactive sites were visualized using a fresh solution of 10 μg of 3,3′-diaminobenzidine tetrahydrochloride (DAB, Sigma-Aldrich, #D5905) in 15 mL of a 0.5 M Tris buffer.

### 2.11. Controls of Specificity

The specificity of each in situ hybridization reaction was checked in repeated trials using sense NUCB probe at a concentration of 2 ng/µL.

The specificity of each immunohistochemical reaction was checked in repeated trials via pre-absorption of primary antibody Nesf-1 (H-003-24; Phoenix Pharmaceuticals) with homologous antigen Nesf-1 (1–45)/Nesf-1, N-terminal (Human) (003-24; Phoenix pharmaceuticals) (up to 50 mg/mL antiserum in the final dilution). Positive controls were made by sections of rat testis [10]. Internal reaction controls were carried out by substituting primary antisera or secondary antisera with 1× PBS or normal serum in the specific step.

### 2.12. Image Acquisition

Fluorescent and light images were observed and analyzed with Zeiss Apotome and processed with Zeiss blue software. Digital raw images were optimized for image resolution, contrast, evenness of illumination, and background using Adobe Photoshop CC 2018 (Adobe Systems, San Jose, CA, USA). Anatomical structures were identified according to the adult *N. furzeri* brain atlas [46]. The immunoreactive cells in the rostral intestinal bulb have been counted. The cell count was carried out manually by using an open source image-processing program (ImageJ). Cells were identified on the basis of their morphological aspect.

## 3. Results

### 3.1. Sequence Analysis and Antibody Specificity

**Figure 1:**
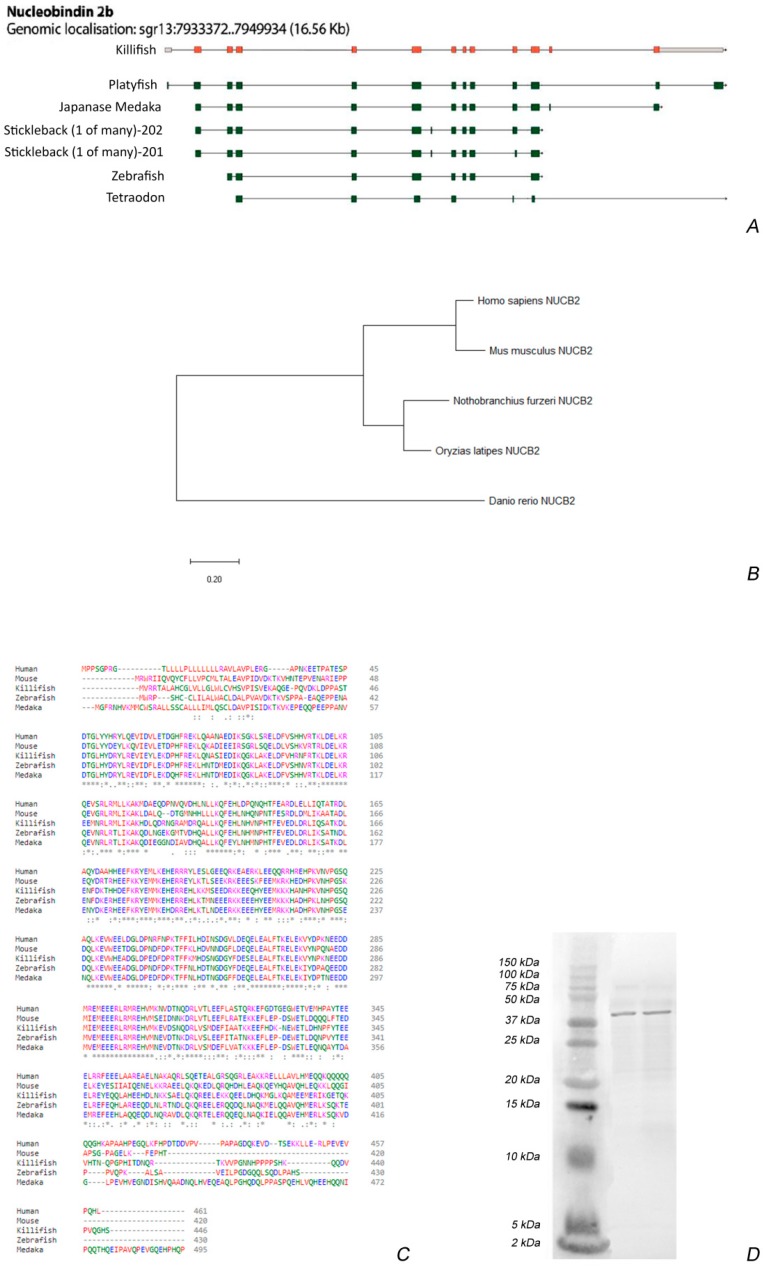
NUCB2B gene structure and Nesfatin–1 peptide analysis: (**A**) NUCB2B intron/exon gene structure of *N. furzeri* and alignment of transcripts from other fish species (downloaded and modified from http://nfingb.leibniz-fli.de/, transcript information for the other fish species can be found at http://www.ensembl.org/). The structure of the two paralogues NUCB2A and NUCB2B were highly similar. In the image, NUCB2B is shown as a template. (**B**) The evolutionary history of NUCB2B peptide sequences was inferred by using the Minimum Evolution method. The phylogenetic tree of amino acid sequences was reconstructed by MEGA X. (**C**) NUCB2B peptide sequence alignments in different species: *Nothobranchus furzeri* (killifish), *Oryzias latipes* (medaka), *Carassius auratus* (goldfish), *Homo sapiens* (human), and *Mus musculus* (mouse). Asterisks mark conserved amino acids (alignment was done with Clustal Omega http://www.ebi.ac.uk/Tools/msa/clustalo/). (**D**) Western blot in the brain of young and old killifish showing an immunoreactive band of about 40 kDa.

The structure of the gene consisted of 13 exons and 12 introns (Figure 1A). Due to the third round of whole-genome duplication in teleost, two paralogues are present in the genome of *N. furzeri*: NUCB2A and NUCB2B. Sequences were obtained from the *Nothobranchius furzeri* Genome Browser–NFINgb (https://nfingb.leibniz-fli.de/): accession number was Nfu_g_1_003870 for NUCB2A and Nfu_g_1_018131 for NUCB2B [34]. Even if the differences between NUCB2A and NUCB2B are very slight, we designed primers to detect only NUCB2B, since it has a higher evolutionary conservation towards mice or humans (Figure 1B). As a result, the primers for generating the ISH probe as well as the qPCR primer bound only to the NUCB2B transcript and the synthetized probe showed 100% identity to NUCB2B (in total 417 nucleotides, transcript position 210 to 626), whereas the identity to NUCB2A was only 36% (in total, 154 nucleotides: transcript position 247 to 341 with 16 mismatches, position 358 to 402 with 6 mismatches, and position 535 to 575 with 5 mismatches).

In *N. furzeri*, like in other fish, Nesf-1 is an 81 amino acid anorexigenic peptide (82 in mammals) encoded in the N-terminal of its translated precursor, nucleobindin-2 (NUCB2). *N. furzeri* Nesf-1 protein sequence showed an overall identity of 78% with Nesf-1 of medaka, 73% with zebrafish, 60% with mouse, and 58% with human (Figure 1C). The antibody specificity was tested by western blot. Currently, there are no commercially available antibodies raised against fish sequences and thus, we employed a polyclonal antibody raised against human Nesf-1 (1–45), recognizing a region highly conserved and corresponding to the bioactive segment of the neuropeptides [47]. The western blot revealed a defined band of −40 kDa in *N. furzeri* brain homogenates of young and old fish (Figure 1D). β-actin, used as an internal marker, showed a band of about 42 kDa.

### 3.2. Expression Levels of NUCB2B mRNA in the Whole Brain and in the Rostral Intestinal Bulb of Young and Old Animals

**Figure 2:**
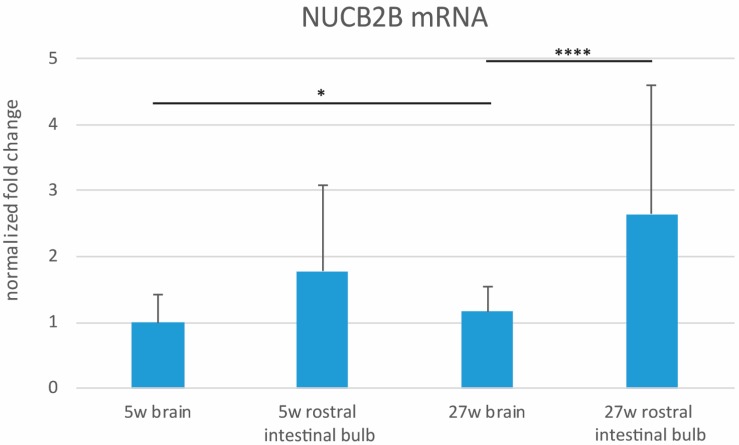
Expression levels of mRNA encoding NUCB2B in the brain and rostral intestinal bulb of young and old *N. furzeri.* The graphic was built on ∆∆CT method and data were normalized to the young brain. T-test and p-value were calculated among young (*n* = 12) and old (*n* = 10) brains and young (*n* = 12) and old (*n* = 10) rostral intestinal bulbs. Furthermore, T-test and *p*-value were also calculated for young brains versus young intestinal bulbs and old brains versus old intestinal bulbs. (*p* brain old versus young = 0.0169; *p* rostral intestine bulb old versus young = 0.0591; *p* young rostral intestinal bulb versus brain = 0.5899; *p* old rostral intestinal bulb versus brain = 0.0001). (*) indicates the level of significance.

The levels of NUCB2B mRNA varied between the two organs and over age. In the brain, we observed a slight augment of NUCB2B levels in old compared to young animals (*p* = 0.0169). In the rostral intestinal bulb, we did not detect a significant variation in the expression levels of NUCB2B in old animals (Figure 2) compared to young (*p* = 0.0591). Furthermore, we questioned which organ could represent the main source of NUCB2B. At sexual maturity, expression levels of NUCB2B were comparable between the rostral intestinal bulb and brain (*p* = 0.5899), although there was a slight increase in the intestinal bulb; whereas at the old stage, NUCB2B mRNA levels were significantly higher in the rostral intestinal bulb than in the brain (*p* = 0.0001) (Figure 2).

### 3.3. Morphological Studies of the Whole Brain of Young and Old Animals

In the present study, the attention was focused on the expression and distribution of either mRNA (ISH) and protein (IHC) onto the areas responsible for the regulation of food intake: the ventral part of the telencephalon and the diencephalon in toto. However, positive labeling was also detected outside the above-mentioned areas, such as olfactory bulbs and dorsal telencephalon in the forebrain, optic tectum, and semicircular tori in the midbrain, which are known to mediate food appetite behavior [32]. Table 1 summarizes positive neurons and fibers localization of NUCB2B mRNA/Nesf-1 protein in young and old brains of *N. furzeri*. The neuroanatomical terminology followed the *N. furzeri* brain atlas [46].

#### 3.3.1. In Situ Hybridization

**Figure 3:**
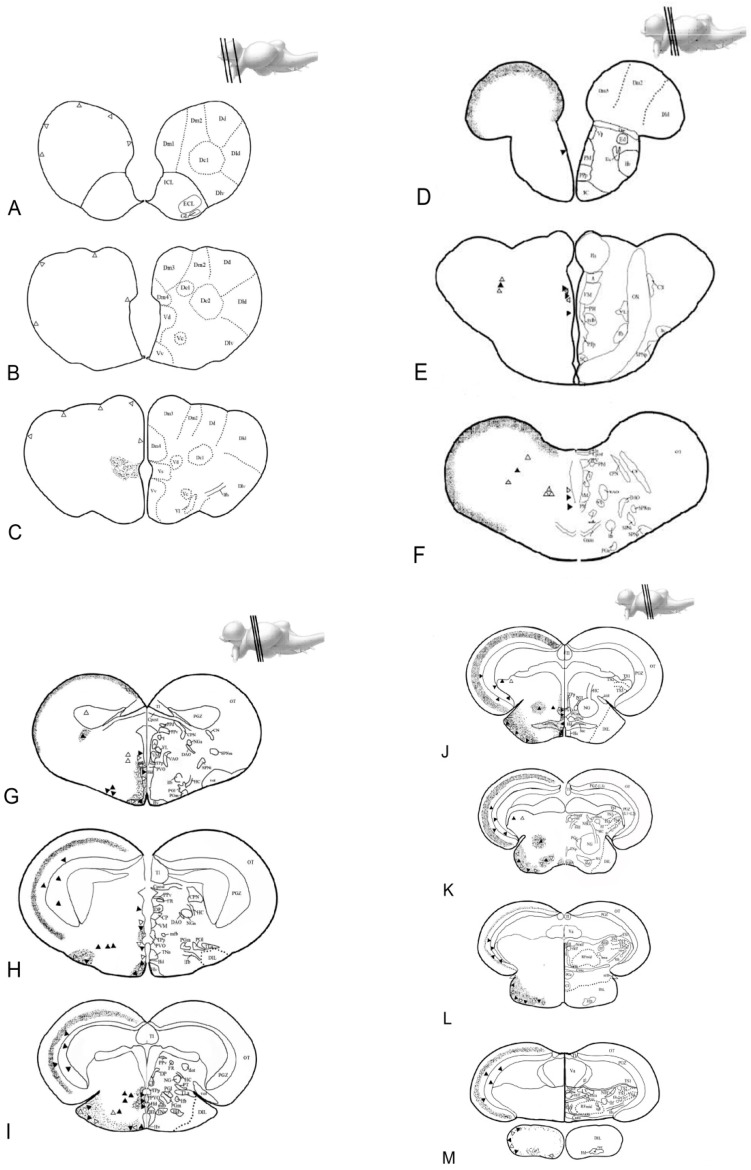
Atlas of NUCB2B gene and Nesf-1 protein distribution: schematic drawings of transversal sections of *N. furzeri* brain [46], specifically referred to (**A**–**F**) forebrain; (**G**–**M**) midbrain. White triangles indicate sites of mRNA positive neurons (ISH); black triangles indicate sites of immunopositive neurons (IHC); small dots indicate immunoreactive fibres (IHC). Scale bar: 200 µm.

**Figure 4:**
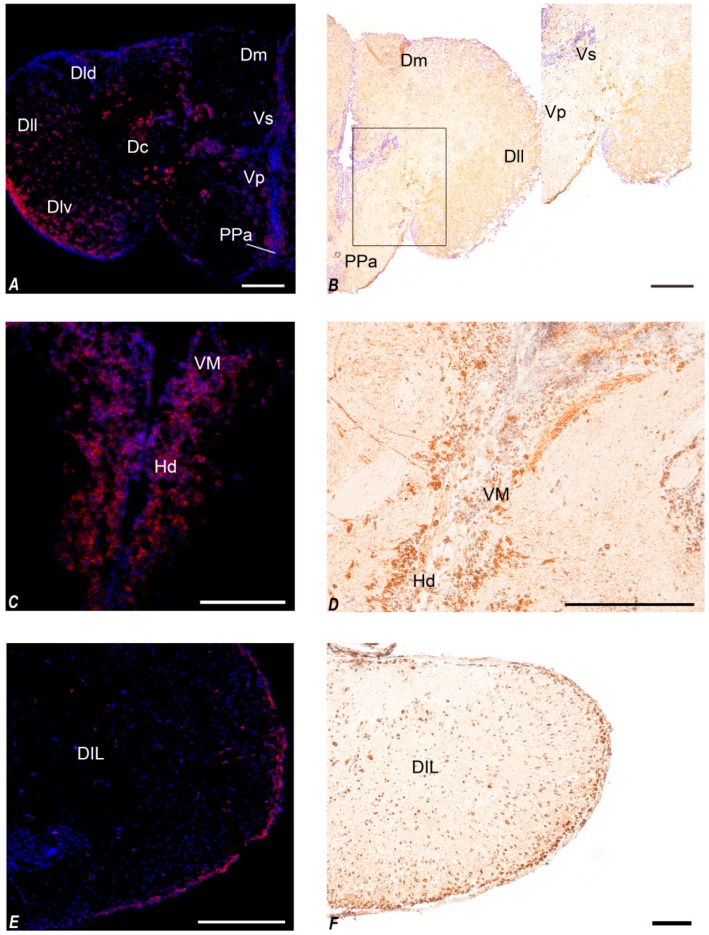
Transversal section showing localization of NUCB2B mRNA and Nesf-1 protein in the young brain of *N. furzeri*. (**A**) NUCB2B expressing neurons in Dc, Dld, Dll, Dlv, Dm, PPa, Vp, and Vs; (**B**) Nesf-1 immunoreactivity (ir) in neurons of Dm, DIL, PPa, Vp, and Vs; (**C**) NUCB2B expressing neurons in VM and Hd; (**D**) Nesf-1 ir in neurons of VM and Hd; (**E**) NUCB2B expressing neurons in DIL; (**F**) Nesf-1 ir in neurons of DIL. Scale bars: A-B-F 100 µm; C-E 200 µm; D 300 µm.

**Figure 5:**
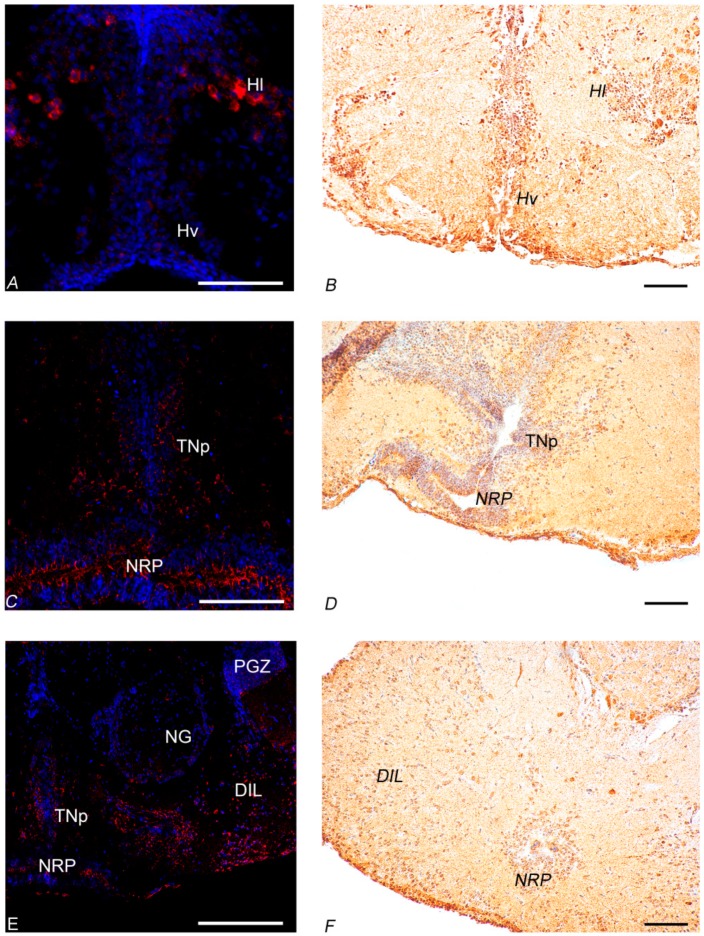
Transversal section showing localization of NUCB2B mRNA and Nesf-1 protein in the brain of old *N. furzeri*. (**A**) NUCB2B expressing neurons in HI and Hv; (**B**) Nesf-1 immunoreactivity (ir) in neurons of HI and Hv; (**C**) NUCB2B expressing neurons in TNp and NRP; (**D**) Nesf-1 ir in neurons of NRP and TNp; (**E**) NUCB2B expressing neurons in DIL, NG, NRP, PGZ, and TNp; (**F**) Nesf-1 ir-neurons of DIL and NRP. Scale bars: A-C-E 200 µm, B-D-F 100 µm.

In the diencephalon of young fish, NUCB2B mRNA was detected in neurons of the cortical nucleus (Figure 3E–G). Positive neurons were displayed in the ventro-medial thalamic nucleus (Figure 3E–H and Figure 4C), in the paraventricular organ (Figure 3G–J), and in the periventricular nucleus of the posterior tubercle (Figure 3G–J and Figure 5C–E) along the ventricle. Labelled neurons were found in the dorsal (Figure 3I,J and Figure 4C), central, and ventral part of the hypothalamus (Figure 3G–J), close to the ventricle. Small and intensely positive neurons were detected in the diffuse inferior lobe of the hypothalamus (Figure 3L,M and Figure 4E). In the diencephalon of old fish, some positive neurons were detected in the periventricular nucleus of the posterior tuberculum. Many packed positive neurons were observed in the hypothalamic region, particularly in the dorsal, ventral, lateral, and caudal parts (Figure 5A). Moreover, many neurons were positively stained in the nucleus of posterior recess (Figure 5C–E), unlike in the young subjects.

**Figure 6:**
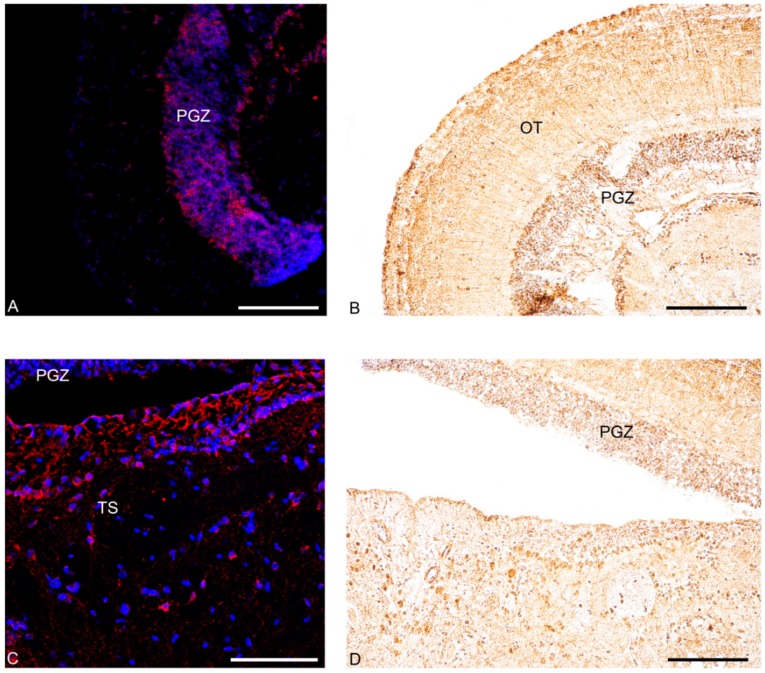
Transversal section showing localization of NUCB2B mRNA and Nesf-1 protein in non-diencephalic regions of *N. furzeri* brain. (**A**,**B**) young fish: (**A**) NUCB2B expressing neurons in PGZ; (**B**) Nesf-1 immunoreactivity (ir) in neurons of PGZ and OT; (**C**,**D**) old fish: (**C**) NUCB2B expressing neurons in PGZ and TS; (**D**) Nesf-1 ir-neurons of PGZ. Scale bars: 200 µm.

In addition, in young fish, positive neurons were detected in non-diencephalic areas: along the margin of the dorsal telencephalon (Figure 3A–C and Figure 4A) and in the periventricular grey zone of the optic tectum (Figure 3G and Figure 6A). Conversely, in old fish, only scarcely positive neurons were detected along the margin of the dorsal telencephalon (Figure 5E and Figure 6C).

#### 3.3.2. Immunohistochemistry

In young fish, immunoreactivity to Nesf-1 protein was found in the ventral telencephalon and diencephalon. In the telencephalic ventral areas, especially in the supracommissural zone, abundant positive ir-fibers were observed (Figure 3C and Figure 4B). In the diencephalon, positive neurons were detected in the magno- and parvo-cellular parts of the preoptic nucleus (Figure 3D–F). Also, ir-neurons enveloped in a tight net of projections were visible in the cortical nucleus (Figure 3E–G), preglomerular nucleus, mainly in its medial part (Figure 3G), pretectal nucleus, as well as in the paraventricular organ (Figure 3G–J). Numerous immune-labelled neurons, with a notable quantity of fibers surrounding, was identified in the ventral, dorsal, and caudal parts of the tuberal hypothalamus. Several stained neurons and fibers were recognized in the thalamic nucleus, both dorsal posterior and ventro-medial parts (Figure 3G–J and Figure 4D). Ir-perikarya were located around the margin of the periventricular nucleus of the posterior tuberculum (Figure 3E–G). Many ir-projections were found in the medial part of the glomerular nucleus (Figure 3J,K). Positive ir-neurons were disseminated in the diffuse inferior lobe of the hypothalamus, especially on the external margin (Figure 3K–M and Figure 4F), with some fibers spread in the medial zone. In non-diencephalic regions, several ir-positive neurons were detected in the optic tectum (Figure 6B), especially packed in the periglomerular grey zone (Figure 6B), with projections towards the external margin (Figure 3G–M). Moreover, weakly ir-neuronal cells were widespread in the different layers of semicircular tori (Figure 3J,K).

Nesf-1 protein distribution did not show notable differences between the young and elderly subjects (Figure 5B,D,F and Figure 6D).

### 3.4. Morphological Studies of the Intestine of Young and Old Animals

**Figure 7:**
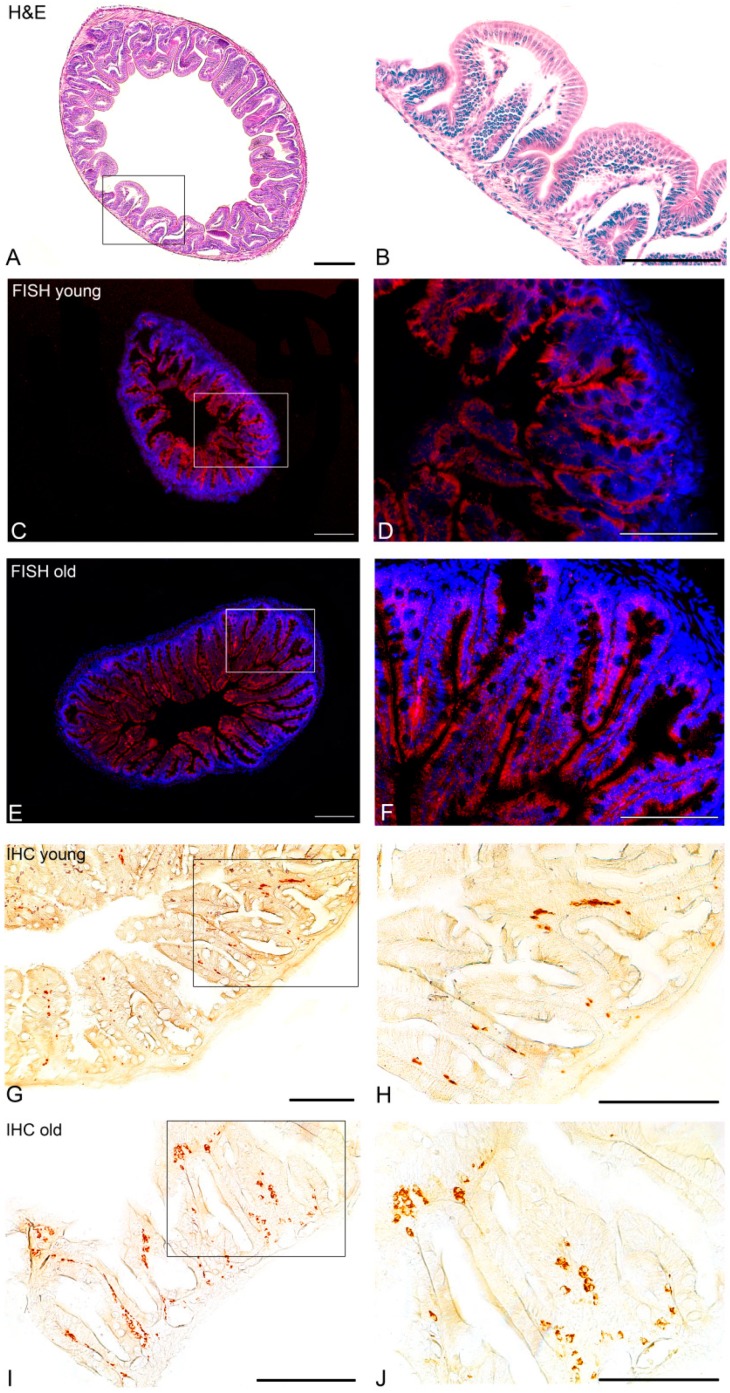
Transversal section showing localization of NUCB2B mRNA and Nesf-1 protein in the rostral intestinal bulb of *N. furzeri*. (**A**) Hematoxylin/eosin staining reveals a simple architecture of a mucosa, submucosa, muscularis externa, and serosa layer; (**B**) Higher magnification of A; (**C**) in young fish NUCB2B mRNA was detected in the lining epithelium; (**D**) Higher magnification of C; (**E**) in old fish, NUCB2B mRNA was detected in the lining epithelium; (**F**) Higher magnification of E; (**G**) in young subjects, Nesf-1-immuonoreactivity (ir) was detected within rounded or flask-shaped cells in submucosa, most of them scattered deep within the folds of the ridges; (**H**) Higher magnification of G; (**I**) in old fish, Nesf-1-ir cells were more abundant in the submucosa and in addition, some of the cells were dispersed along the apical regions of the ridges; (**J**) Higher magnification of I. Scale bars: A-C-E 100 µm, B-D-F-H-I-J 300 µm, G 200 µm.

*N. furzeri*, as a Cyprinodontiformes species, belongs to the group of agastric fish in which the intestine transits directly from the esophagus [48]. In adults, the intestine is folded into three sections: the rostral intestinal bulb, mid-intestine, and caudal intestine. We focused our analysis on the rostral intestinal bulb, which is known to likely serve a similar function to the mammalian stomach [49]. Sagittal sections of the rostral intestinal bulb reveal a simple architecture of a mucosa, submucosa, muscularis externa, and serosa layer (Figure 7A,B). The intestinal mucosa consists of columnar shaped enterocytes (Figure 7B). We observed that the intestine surface is covered by ridges that are oriented circumferentially across the intestine axis (Figure 7A,B). These ridges in the cross-section resemble the spatially separate villi in the mouse or human small intestine.

#### 3.4.1. In Situ Hybridization

In the rostral intestinal bulb of young fish, NUCB2B mRNA was detected in the lining epithelium. Signal was restricted to the columnar shaped enterocytes of the apical regions of the ridges (Figure 7C,D). NUC2B mRNA distribution did not show notable differences between young and old subjects (Figure 7E,F).

#### 3.4.2. Immunohistochemistry

Cross sections of the rostral intestinal bulb of young fish showed Nesf-1 positive rounded or flask-shaped cells in submucosa, mainly scattered deep within the folds of the ridges (Figure 7G,H). In old fish, Nesf-1 ir-cells were more abundant in the submucosa and, in addition, some of the cells were dispersed along the apical regions of the ridges (Figure 7I,J). The number of positive cells did not reveal differences between the young and old animals (Figure S1).

## 4. Discussion

In the present survey, we characterized NUCB2B/Nesf-1 in the brain and in the mammalian stomach equivalent structure of *N. furzeri* and reported, for the first time, its regulation upon aging in a teleost species.

In fish, two isoforms of NUCB2 (NUCB2A and NUCB2B) exist, which presumably arose due to the teleosts-specific whole genome duplication known as 3R (third round of genome duplication) [50]. Also, in *N. furzeri* [34], two paralogues exist: NUCB2A and NUCB2B. Here, we determined that NUCB2A and NUCB2B gene sequences in killifish are highly similar to NUCB2 from other fish and mammals. This suggests that NUCB2 and its paralogues are highly conserved genes; therefore, Nesf-1 (a product of proteolytic processing of NUCB2 protein) has a similar aminoacid sequence among vertebrates. In fish, the proposed prohormone convertase cleavage site (Lys-Arg) is ubiquitously conserved in both NUCB2A and NUCB2B genes, suggesting that the putative Nesf-1 peptide can be cleaved from the larger NUCB2 precursor [14,15]. We analyzed the distribution of NUCB2B mRNA in the whole brain and the transcript was mainly detected in the hypothalamic nuclei, as previously reported in mammals [3,6,51,52,53,54], as well as in other vertebrate species such as frogs [55] and goldfish [14]. In young animals, neurons expressing NUCB2B mRNA were localized in the cortical nucleus, ventro-medial thalamic nucleus, paraventricular organ, and diffuse inferior lobe of the hypothalamus. We also studied Nesf-1 protein distribution by a commercial antibody, detecting the precursor Nesf-1 (1–82). Western blot analysis on the whole brain of *N. furzeri* reveals that the protein is expressed with the expected molecular weight of about 40 kDa, as also reported in goldfish [14] and mammals [51]. In *N. furzeri*, immunoreactivity to Nesf-1 was detected in the hypothalamic area, both in neuronal perikarya and fibers of young and old animals. Some areas, i.e., paraventricular organ and the diffuse inferior lobe of the hypothalamus, displayed positive neurons in ISH and IHC. Interestingly, mRNA expression and protein distribution were also detected in non-diencephalic areas, specifically in the telencephalon, optic tectum, and semicircular tori of both young and old animals. In mammals, Nesf-1 immunoreactivity was observed in non-diencephalic areas as well, including the nucleus of tractus solitarius, another brain region implicated in the regulation of feeding [6,51,52]. Our results in the brain of *N. furzeri* agree with previous observations in goldfish, where immunohistochemical studies showed the presence of Nesf-1-like ir within the hypothalamus and preoptic areas [14]. In teleost fish, both telencephalon and optic tectum are known to be involved in the control of appetite [56]. For example, electrical stimulation of either the ventral telencephalon, secondary gustatory nucleus, or optic tectum induces enhanced feeding behavior [57], whereas feeding behavior is depressed by olfactory tract lesions [57,58]. It might be possible that the presence of NUC2B/Nesf-1 in non-diencephalic areas of *N. furzeri* brain implicates that these areas could also be involved in the regulation of feeding in this species and/or several other homeostatic systems [59].

The primary source of NUCB2/Nesf-1 is currently unknown in vertebrates. In mammals, the expression level of NUCB2 in the stomach was found to be 10-fold higher than the levels of the brain [11], suggesting a prominent role for this organ in the synthesis and secretion of NUCB2/Nesf-1. As the most common teleost fish used as an animal model, such as zebrafish [60], medaka [48], or goldfish [14], *N. furzeri* also has no proper stomach, which is different from what has been reported in a previous paper [31]. According to Smith [61], a true stomach always has mucosal glands producing hydrochloric acid juice and can be closed by a sphincter at its caudal end. In addition, the loss of the stomach phenotype is accompanied by the loss of pepsinogen and gastric proton pump genes [48]. In agastric fish, the intestine transits directly from the esophagus and usually it can be divided into three sections (anterior, mid-intestine, and caudal intestine), where the dilatation of the first section of the intestine (named the rostral intestinal bulb or J-loop) is considered homologous to the mammalian stomach [48]. For the first time, we described the rostral intestinal bulb of *N. furzeri*. The wall of the intestine showed the superposition of four tunicae, which is typical of vertebrates. Genome analysis indicates the absence of pepsinogen gene in *N. furzeri*. In the rostral intestinal bulb, NUCB2B mRNA was detected in the epithelial cells lining while Nesf-1 immunoreactivity was mainly observed in the submucosa. The morphological pattern observed in the intestine of *N. furzeri* agree with previous observations in zebrafish, where NUCB2/nesfatin-1-like ir was detected in most of the cells scattered deep within the folds of the villi of J-loop [16], and disagree with a previous study of goldfish, where Nesf-1-like ir was only detected in enteroendocrine like cells of the intestinal villi [14]. It is likely that NUCB2 concurs to regulate the high turnover of epithelial intestinal cells. Future studies are mandatory to explore the peripheral role of NUCB2B/Nesf-1in *N. furzeri*, taking into consideration that currently the receptor(s) mediating the regulatory mechanisms of actions of Nesf-1 remain unknown [47].

Up to now, this is the first study reporting the regulation of NUCB2/Nesf-1 during aging in a vertebrate model. Interestingly, we document the increase of NUCB2B during aging, with the highest expression at the peripheral level. This could be related to an energy drive failure leading to anorexic phenotype, which indeed is well described and typically occurs during aging [62]. There are enough demonstrations available to indicate that nucleobindin and its encoded peptides have a pleiotropic role in cell biology, such as inhibition of proliferation and enhancing apoptosis [63]. In this context, it is relevant to specify that in the current genome annotation of *N. furzeri*, neither ghrelin nor leptin b were annotated, which are highly conserved during vertebrate evolution. With the exclusion of any annotation error, it is likely that that they are evolutionarily lost. This will be in line with the presence of an anorexic phenotype in old *N. furzeri* and it would elucidate a new role of nesfatin as an independent anorexigenic player in this short-living species.

These results, obtained in a phylogenetically distant vertebrate, highlight the growing evidence in support of a role for Nesf-1 as a novel brain-gut regulatory peptide. The communication between the central nervous system and the gastrointestinal tract plays a fundamental role in the regulation of food intake and energy balance, modulating short-term satiety and hunger responses. This axis also has a role in the regulation of blood glucose levels and adipocyte function [64]. The gut-brain axis has both neuronal and humoral components that convey information to the key brain regions involved in the homeostatic regulation of feeding, located mainly in the hypothalamus (including the arcuate nucleus) and brainstem (including the nucleus tractus solitarius) [65]. A number of gut hormones have been identified in the gastrointestinal system, including Nesf-1 [66], which are released from enteroendocrine cells. In addition to the coordination of the digestive process, they also relay information regarding the current state of energy balance, exerting endocrine effects on other organ systems, particularly the brain, where some have also been found to exist as neurotransmitters [67].

## 5. Conclusions

In conclusion, the present work provides the first evidence for the occurrence of NUCB2B/Nesf-1 in *N. furzeri* and to the best of our knowledge, it is the first description of aging regulation reported in any vertebrate. We consider that the experimental modulation could contribute to validate the African turquoise killifish in clinical and preclinical studies. Indeed, in previous work [28], we used 96 h of fasting as a paradigm to better evaluate the regulation of neuropeptides involved in food intake. In that experimental design, 96 h of fasting represents a metabolic stimulus in this species, since we have demonstrated that it is able to activate neurons (pS6 marker), although in the brain of old animals, very few neurons were activated, suggesting that the metabolic stimulus needs to be more intense in old organisms with physiologically low metabolic rates. Based on these evidences, we are planning a similar experiment with a longer starvation period to evaluate the activated metabolic pathways at central and peripheral levels.

## Figures and Tables

**Table 1:** Summary of positive perikarya and fibers localization of NUCB2B mRNA and Nesf-1 protein in the forebrain of young and old *N. furzeri*.

	Young Brain			Old Brain		
	mRNA	Protein		mRNA	Protein	
	Perikarya	Perikarya	Fibers	Perikarya	Perikarya	Fibers
**Telencephalon**						
Dorsal Telencephalon						
Sopracommissural Zone of the Ventral Telencephalon (Vs)			+			+
Central Part of the Ventral Telencephalon (Vc)			+			+
**Preoptic area**						
Preoptic Nucleus, Parvocellular Part (PPp)		+			+	
Cortical Nucleus (CN)	+	+			+	
Lateral Preglomerular Nucleus (PGI)		+			+	
Medial Preglomerular Nucleus (PGm)		+			+	
**Tuberal hypothalamus**						
Dorsal Hypothalamus (Hd)	+	+	+	+	+	+
Ventral Hypothalamus (Hv)	+	+	+	+	+	+
Caudal Hypothalamus (Hc)	+	+		+	+	
Periventricular Nucleus of Posterior Tuberculum (TPp)			+	+		+
Glomerular Nucleus (NG)		+	+		+	+
Nucleus of Posterior Recess (NRP)	+		+	+		+
Diffuse Inferior Lobe of Hypothalamus (DIL)	+	+	+		+	+
**Posterior tubercle**						
Paraventricular Organ (PVO)	+	+	+		+	+
**Thalamus**						
Dorsal Posterior Talamic Nucleus (DP)		+			+	
Ventro-Medial Talamic Nucleus (VM)	+	+			+	

## Supplementary Materials

The following are available online at https://www.mdpi.com/2077-0383/9/1/103/s1, Figure S1: NESF-1 positive cells in the submucosa of the rostral intestinal bulb of young and old animals. Cell count was carried out manually on 7 consecutive sections by using an open source image-processing program (ImageJ). Cells were identified on the basis of their morphological aspect. The graphical analysis was produced by Excel and did not reveal any significance difference between the two age points studied, Table S1: Ensemble Species Code, Table S2: Summary of primer pair sequences used for Real Time PCR (RT- PCR) and in situ hybridization (ISH).

## Abbreviations

*Dc*	central zone of dorsal telencephalon
*Dld*	dorso-lateral zone of dorsal telencephalon
*Dll*	latero-lateral zone of dorsal telencephalon
*Dlv*	ventro-lateral zone of dorsal telencephalon
*Dm*	medial zone of dorsal telencephalon
*DIL*	inferior lobe of hypothalamus
*Hd*	dorsal hypothalamus
*Hl*	lateral hypothalamus
*Hv*	ventral hypothalamus
*NG*	glomerular nucleus
*NRP*	nucleus of posterior recess
*OT*	optic tectum
*PGZ*	periventricular grey zone of Optic Tectum
*PPa*	anterior preoptic nucleus
*TNp*	posterior tuberal nucleus
*TS*	semicircular tori
*VM*	ventro-medial thalamic nucleus
*Vp*	posterior zone of ventral telencephalon
*Vs*	supracommisural zone of ventral telencephalon
