# Supplementary material for: Ontogenetic Pattern Changes of Nucleobindin-2/Nesfatin-1 in the Brain and Intestinal Bulb of the Short Lived African Turquoise Killifish
Source: J Clin Med. 2019 Dec 31;9(1):103. doi: 10.3390/jcm9010103 (PMC7019235; doi:10.3390/jcm9010103)
Supplement: Supplementary file 1 [file jcm-09-00103-s001.pdf]

Table S1. Ensemble Species Code.

Species	Name of Gene	Ensemble Code
Xiphophorus maculatus	nucleobindin 2b	ENSXMAG00000012412
Oryzias latipes	nucleobindin 2	ENSORLT00000001613
Gasterosteus aculeatus	NUCB2 (1 of many)-201	ENSGACG00000006759.1
Gasterosteus aculeatus	NUCB2 (1 of many)-202	ENSGACT00000009029
Danio rerio	nucleobindin 2b	ENSDARG00000036291
Tetraodon	nucleobindin 2	ENSTNIT00000005819

Table S2. Summary of primer pair sequences used for Real Time PCR (RT- PCR) and *in situ* hybridization (ISH).

<table>
<tr><th>Gene</th><th>RT-PCR primer sequence</th><th>Product size lenght</th><th>Annealing temperature</th></tr>
<tr><td>NUCB2_fw</td><td>ACT GTG GGC TGG TCC TAC TG</td><td rowspan="2">150 bp</td><td rowspan="2">60 °C</td></tr>
<tr><td>NUCB2_rev</td><td>CTT CCC TGA GGT AAC GGT CA</td></tr>
<tr><td>TBP_fw</td><td>CGGTTGGAGGGTTTAGTCCT</td><td rowspan="2">100 bp</td><td rowspan="2">60 °C</td></tr>
<tr><td>TBP_rev</td><td>GCAAGACGATTCTGGGTTTG</td></tr>
<tr><th>Gene</th><th>ISH primer sequence</th><th>Product size length</th><th>Annealing temperature</th></tr>
<tr><td>NUCB2_fw</td><td>CCA CCA GCG AGC ACT GAC ACC</td><td rowspan="2">416 bp</td><td rowspan="2">60 °C</td></tr>
<tr><td>NUCB2_rev</td><td>GGT AAT ACG ACT CAC TAT AGGATA CCT CTT GAA CTC ATC ATG</td></tr>
</table>

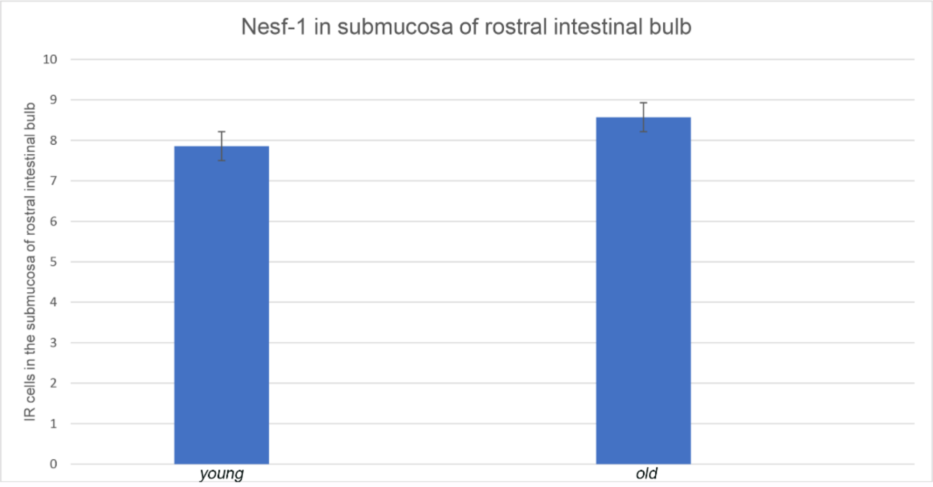

Figure S1. NESF-1 positive cells in the submucosa of the rostral intestinal bulb of young and old animals. Cell count was carried out manually on 7 consecutive sections by using an open source image-processing program (ImageJ). Cells were identified on the basis of their morphological aspect. The graphical analysis was produced by Excel and did not reveal any significance difference between the two age points studied.
